# Transcriptomic and microstructural analyses in *Liriodendron tulipifera* Linn. reveal candidate genes involved in nectary development and nectar secretion

**DOI:** 10.1186/s12870-019-2140-0

**Published:** 2019-12-02

**Authors:** Huanhuan Liu, Jikai Ma, Huogen Li

**Affiliations:** Co-Innovation Center for Sustainable Forestry in Southern China, Nanjing Forestry University, 159 Longpan Road, Nanjing, 210037 Jiangsu China

**Keywords:** Nectary, Transcriptome, Morphology, *Liriodendron*, Secretion

## Abstract

**Background:**

Nectar is a major floral attractant and reward for insects that ensures pollination. *Liriodendron*, a genus of the Magnoliaceae family, includes only two relict species, *L. chinense* and *L. tulipifera*, which are considered “basal angiosperms” according to plant evolutionary history. The flowers of *Liriodendron* plants are insect pollinated and secrete nectar to attract pollinators. To date, the morphology and anatomy of nectaries, the mechanism of nectar secretion and the molecular mechanism of nectary development in *Liriodendron* remain poorly understood.

**Methods:**

In this study, we examined the nectary surface cells and change in starch in *L. tulipifera* by using scanning electron microscopy and periodic acid-Schiff techniques to select appropriate samples for subsequent research. Transcriptome sequencing was of the top and middle parts of immature nectaries and the middle part of mature and postsecretory nectaries in *L. tulipifera* was performed. We evaluated the expression profiles of 21 DEGs that are closely related to nectary development and nectar secretion for real-time quantitative PCR analysis.

**Results:**

*L. tulipifera* nectaries are starch-storing nectaries and are located in the top and middle parts of *L. tulipifera* petals. After analyzing the RNA-seq data, we obtained 115.26 Gb of clean data in 12 libraries and mapped the results to the *L. chinense* reference genome with 71.02–79.77% efficiency. In total, 26,955 DEGs were identified by performing six pairwise comparisons. The flavonoid biosynthesis, phenylpropanoid biosynthesis, anthocyanin biosynthesis and starch and sucrose metabolism pathways were enriched and related to nectar secretion and pigment change. We identified 56 transcription factor families, and members of the *TCP*, *Trihelix*, *C2H2*, *ERF*, and *MADS* families changed dynamically during nectary development. Moreover, to further verify the accuracy of the RNA-seq results, we validated the expression profiles of 21 candidate genes.

**Conclusions:**

We evaluated the nectary development and secretion processes comprehensively and identified many related candidate genes in *L. tulipifera*. These findings suggest that nectaries play important roles in flavonoid synthesis and petal color presentation.

## Background

The original attractant for insects to insect-pollinated plants was pollen; however, nectar, an inexpensive foodstuff, was generated as an alternative to pollen, and then, the nectary was formed [1]. Owing to their diversity in position and structure, nectaries are classified into different categories [2]. According to their position, nectaries can be classified into two types: floral and extrafloral nectaries. *Pteridium aquilinum* is an ancient extrafloral plant with nectaries on its fronds [3–5]. According to their structure, nectaries can be divided into two groups: structural and nonstructural nectaries [6, 7]. The structure of most angiosperm nectaries contains three major components: epidermis, specialized parenchyma cell and vascular bundles [8, 9]. During nectary development and nectar secretion in most plants, starch grains accumulate and then degrade. Therefore, according to whether starch undergoes regular dynamic changes, nectaries can also be divided into two groups: starch-storing nectaries and non-starch-storing nectaries [10]. Thus far, the anatomy and taxonomy of nectaries have been comprehensively studied, and substantial progress has been made. However, research on the biochemistry and molecular biology of nectaries is delayed.

Currently, research is focused more on the molecular mechanisms of floral nectaries than on the anatomy and taxonomy of floral nectaries. Several studies on model plants have found a key gene regulating nectary development: *CRABS CLAW* (*CRC*). *CRC* is a transcription factor (TF) containing a zinc finger and a helix-loop-helix domain and is mostly expressed in the carpels and nectaries of mutant and wild-type *Arabidopsis thaliana* [11]. *CRC* is negatively regulated by the A and B organ identity genes and is regulated independently of C class genes according to research on ectopic carpels of floral homeotic mutants [11]. Interestingly, the *crc-1* and *crc-2* mutants both lack nectaries. Baum et al. proposed that the nectary is independent of any floral organ identity genes in *A. thaliana* but associated with genes in the “third whorl” domain: *LEAFY* (*LFY*) and *UNUSUAL FLORAL ORGANS* (*UFO*) [12]. *CRC* is conservatively expressed in the nectaries of several core eudicot plants and is required for nectary development in both rosids and asterids, but *CRC* expression is not found in the nectaries of basal eudicot plants [2]. In addition, *BLADE-ON-PETIOLE1* (*BOP1*) and *BOP2* can also promote nectary development. Interestingly, nectaries are present but abnormal in the *bop1*/*bop2* double mutant, while the *bop1/bop2/crc* triple mutant lacks nectaries [13]. The *Arabidopsis* nectary transcriptome was analyzed, and *CRC*, *A*GL5/*SHATTERPROOF2* (*SHP2*), *AGL1*/*SHP1*, *AGAMOUS* (*AG*) and *APETALA2* (*AP2*) showed nectary-enriched expression profiles [14]. The *CELL WALL INVERTASE4* (*CWINV4*) gene is highly expressed in nectaries and is required for nectar secretion, but the *cwinv4* mutant cannot produce nectar in *Arabidopsis* [15]. The TF *MYB305* is strongly expressed in ornamental tobacco nectaries and regulates the expression of flavonoid metabolic and nectar proteins [16]. When *MYB305* is knocked down, starch accumulation and nectar production are reduced significantly in tobacco nectaries [17]. Other *MYB* TFs, such as *MYB21* and *MYB24*, regulate *Arabidopsis* nectary development and function and volatile sesquiterpene production via cooperation with the jasmonate synthesis pathway [18, 19]. *PIN6* and *MYB57* are nectary-associated genes, and the former is positively regulated during nectar production and required for a proper auxin response in *Arabidopsis* [20].

Although many achievements in research on nectary development and nectar secretion have been made in herbaceous plants, research on this topic in woody plants is lacking. *Liriodendron* species are insect pollinated and secrete nectar to attract pollinators. As “basal angiosperms”, Magnoliaceae species occupy a critical evolutionary position, and a genus of Magnoliaceae, *Liriodendron*, has only two relict species: *L. chinense* and *L. tulipifera* [21–26]. The nectar production of *L. tulipifera*, a nectariferous plant, is greater than that of *L. chinense* [27, 28]. The nectaries in *L. tulipifera* are located in petals. Furthermore, 42 nectar proteins have been identified [28]. However, the exact morphology and anatomical structure during floral nectary development, the mechanism of nectar secretion, and the molecular mechanisms of nectary development and nectar secretion are unclear. In this study, ultrastructural observations of nectaries and dynamic changes in starch in *L. tulipifera* were performed by scanning electron microscopy (SEM) and periodic acid-Schiff (PAS) techniques to select appropriate samples for further research. Then, high-throughput sequencing was performed to identify the genes and pathways potentially involved in the processes of nectary development and nectar secretion in *L. tulipifera*.

## Methods

### Plant materials

All plant materials were collected from 26-year-old trees in a provenance trial plantation of *Liriodendron* located in Zhenjiang, Jiangsu Province (119°13′20″E, 32°7′8″N). The plantation was established in 1994 and contained 12 provenances of *L. chinense* and 5 provenances of *L. tulipifera* [29]. One *L. tulipifera* tree (seed source: Georgia, USA) was used as the sample plant to collect petals at intervals of 3 to 7 days from February to May in 2018.

### Observation of dynamic changes in nectary surface morphology and starch

We collected petals from stage 1 to stage 6. Each petal was divided into three parts according to its color and then fixed in formalin-acetic acid-alcohol (FAA) solution for SEM and PAS techniques. The fixed material was dehydrated in an ethanol series, dried to the CO_2_ critical point by an Emitech K850 critical point dryer (Emitech, Canterbury, UK), gold plated by an Edwards E-1010 gold ion sputter coater (Hitachi, Tokyo, Japan) and observed with an FEI Quanta 200 scanning electron microscope (FEI, Eindhoven, the Netherlands). Some fixed petals were dehydrated in an ethanol series, transparentized in a xylene series, infiltrated in paraffin, and embedded in paraffin wax. The samples were sliced into several 3–4 μm thick slices with a Leica RM2145 rotary microtome (Leica Microsystems, Wetzlar, Germany) and stained via the PAS technique following the manufacturer’s instructions (Solarbio, Beijing, China). Then, the samples were sealed with neutral balata and observed with an Axioscope A1 fluorescence microscope (Carl Zeiss, Jena, Germany).

### RNA extraction, cDNA library construction, and Illumina sequencing

Based on the results of anatomical observation and PAS staining, four different samples of petals were prepared from *L. tulipifera*: immature nectary (green-white color, middle section, stage 2), immature nectary (green, top section, stage 2), mature nectary (yellow color, middle section, stage 3), and postsecretory nectary (orange-red, middle section, stage 5) (Table 1). The samples were quickly frozen in liquid nitrogen and stored at −80°C in a refrigerator. Total RNA was extracted using an RNAprep Pure Kit (Tiangen, China) following the manufacturer’s instructions. RNA quality was checked using a NanoDrop 1000 spectrophotometer (Thermo Fisher Scientific, Wilmington, DE). The cDNA libraries were sequenced on the Illumina Nova platform (California, USA) with a 125-bp paired-end read length at Biomarker Technologies (Beijing, China).

**Table 1:** Sample description and numbers of RNA-seq reads

Developmental Stage	Sample Description	Sample ID	Total Reads	Mapped Reads	% ≥ Q30
Stage 2	top; green; immature nectary	S2T	56.2 M	42.7 M (76.09%)	94.60%
			71.6 M	52.3 M (73.06%)	94.42%
			53.3 M	37.8 M (71.02%)	94.28%
Stage 2	middle; green-white; immature nectary	S2M	81.2 M	62.6 M (77.07%)	94.72%
			73.2 M	56.8 M (77.53%)	94.72%
			69.5 M	51.1 M (73.47%)	94.57%
Stage 3	middle; yellow; mature nectary	S3M	72.8 M	56.4 M (77.52%)	94.79%
			66.8 M	51.9 M (77.72%)	94.55%
			57.3 M	45.5 M (79.32%)	94.97%
Stage 5	middle; orange-red; postsecretory nectary	S5M	56.8 M	43.9 M (77.28%)	94.90%
			56.9 M	43.0 M (75.58%)	93.85%
			58.0 M	46.3 M (79.77%)	94.69%

*M* Millions

### Data analysis for RNA-seq experiments

The raw reads were cleaned by removing adapter sequences and low-quality reads [30]. Clean data were mapped to the *L. chinense* genome sequence (NCBI, Bio Project, PRJNA418360) with HISAT2 (v2.0.5, [31, 32]). The NR, Swiss-Prot, Gene Ontology (GO), Clusters of Orthologous Groups (COG), Eukaryotic Orthologous Groups (KOG), Kyoto Encyclopedia of Genes and Genomes (KEGG), and Pfam databases were used [33–39].

### Identification, annotation and enrichment analysis of differentially expressed genes

To identify differentially expressed genes (DEG) related to the development of floral nectaries, we mapped clean reads to the transcriptome assemblies for each sample using RNA-seq by expectation maximization (RSEM) and for two different samples by the DESeq method [40, 41]. A false discovery rate (FDR) of 0.01 and a fold change (FC) of 2 were chosen as the thresholds for DEG identification. Then, the identified DEGs were used for GO and COG classification and KEGG pathway enrichment analysis.

### Real-time quantitative PCR validation

To verify the RNA-sequencing results, we evaluated the expression profiles of 22 DEGs that were closely related to nectary development and nectar secretion and chose *LcActin97* as a reference gene for real-time quantitative PCR (RT-qPCR) analysis. All primers for RT-qPCR were designed by Oligo7 software and are listed in Additional file 1: Table S1. RT-qPCR was performed using a TB Green Premix Ex Taq Kit (Takara, Shiga, Japan) and run on an ABI StepOne plus thermal cycler (Applied Biosystems, California, USA). The relative expression of candidate DEGs was analyzed using the 2^−ΔΔCT^ method.

## Results

### Surface morphology of *L. tulipifera* nectaries

**Fig. 1:**
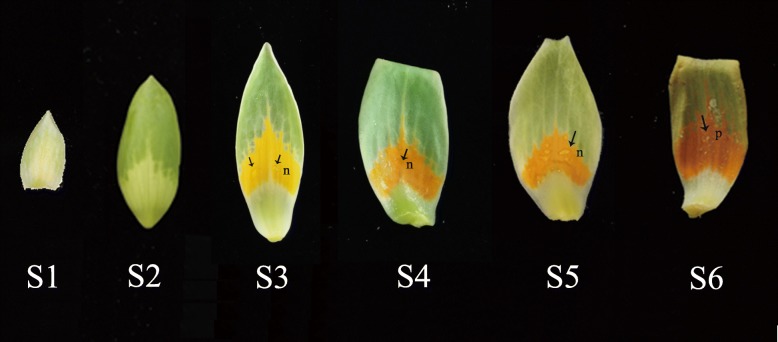
The development process of petals in *Liriodendron*. S1: stage1, all floral organs elongated; S2: stage2, the petal was over twice the size of the petal in stage 1 and the base was fleshy and white; S3: stage3, each petal present three colors obviously, with green on the top, yellow in the middle part, and white at base; S4: stage4, the petal color changed from yellow to orange in the middle part; S5: stage 5, the color of the middle part deepened; S6: stage 6, the petal fall; n: nectar; p: pllen

According to the floral organ traits, we divided petals into six stages (Fig. 1). After dormancy, all floral organs began elongating (stage 1). One month later, the petals were over twice the size of the petals in stage 1, and the base was fleshy and white (stage 2). Each petal had three colors, with green on the top, yellow in the middle part, and white at the base. At the study site, the first flower of *L. tulipifera* appeared in April (stage 3). The blossoming stage lasted approximately 20 days (stage 4). During stage 4, the petal color in the middle part changed from yellow to orange. There was an abundance of nectar on the surface of petals from stage 3 to stage 4. As the petals transitioned to stage 5, the color of the middle part deepened, while nectar production declined. In the last stage, the petals began to fall, and floral development was completed (stage 6).

**Fig. 2:**
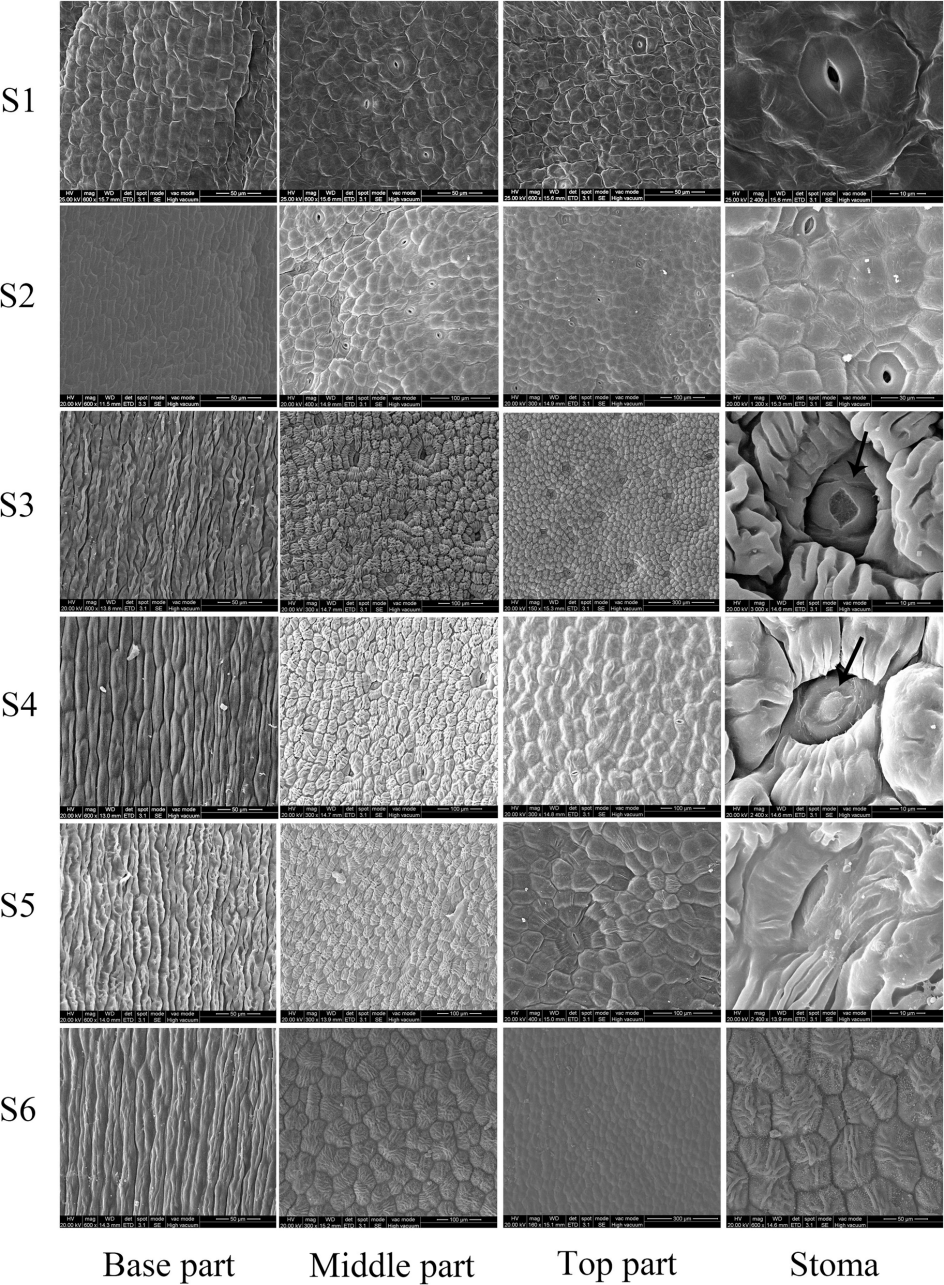
The epidermal cells of petals during flower development in *L. tulipifera*. The arrows mean secretions from the petal

Then, to understand the process of nectar production and the differences between the three parts of the petals in *L. tulipifera*, the surface morphology of the petals was observed via SEM. As shown in Fig. 2, we recorded changes in the epidermal cell morphology of the three parts and stomas from stage 1 to stage 6. The basal part had only a few stomas at stage 1, and the epidermal cell morphology was irregular. Then, there were no changes in the stomas or epidermal cells observed from stage 2 to stage 6 (Fig. 2). We speculated that the main functions of the basal part are to support the petals and transport nectar to the middle and the top parts rather than to secrete nectar. The epidermal cell morphologies and abundance of stomas in the middle and top parts were similar, but the traits of the former were more evident than those of the latter in *L. tulipifera*. At stage 1, the epidermal cells were flat and irregular, and the stomas were normal. At stage 2, the epidermal cells became plump and polygonal, and the stomas were sunken slightly. From stage 3 to stage 5, the morphologies of the epidermal cells and stomas changed substantially. At stage 3, interestingly, the stomas were modified, open and full of a liquid (nectar) that was prepared for secretion. Next, we observed nectar, which was secreted from modified stomas with deep depressions. When stage 5 and stage 6 commenced, the stomas closed, degenerated and stopped secreting nectar. According to the stomal and epidermal cell morphologies, the nectaries were mature at stage 3, and the nectar began to be secreted by modified stomas at stage 4. The epidermal cells were severely corrugated and tightly ordered. The modified stomas opened at stage 3 and closed at stage 5 and stage 6. At stage 6, the cells were flat again. Based on these results, the nectaries are located in the middle and top parts of the petals, and the nectaries secrete nectar at stage 3.

### Dynamic changes in starch in *L. tulipifera*

**Fig 3:**
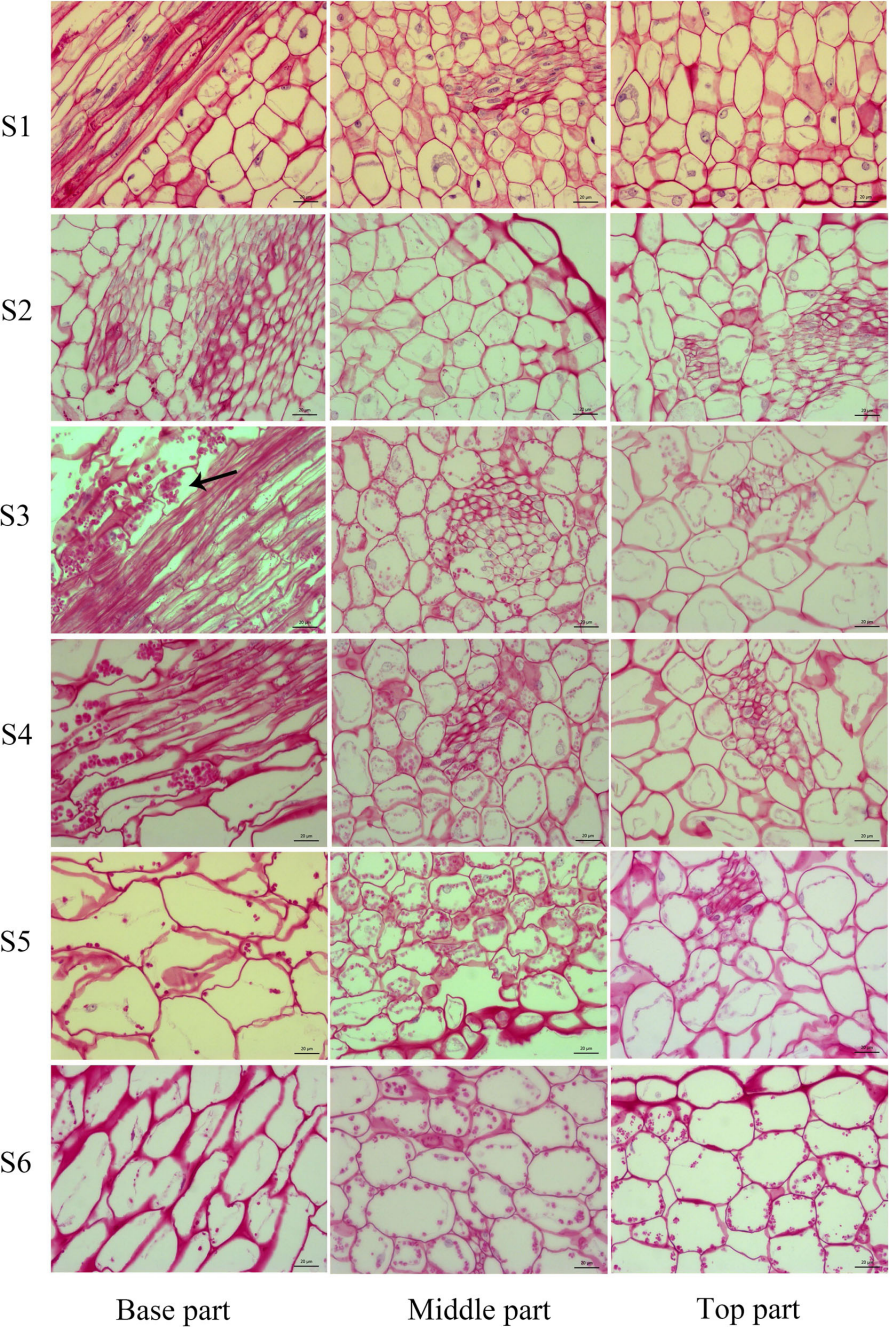
The dynamic changes in starch during flower development in *L. tulipifera*. The arrow means starch grain in the petal

To further investigate the mechanism by which nectar is secreted and whether the nectaries in *L. tulipifera* are starch-storing nectaries, we measured the change in starch in the three petal parts during six stages using the PAS method. At stage 1, there were no starch grains in the petals (Fig. 3). Afterwards, the basal part had visible starch grains, and the middle and top parts began to accumulate starch. Notably, there was an abundance of starch grains in the whole sepal at stage 3, especially in the basal part. The number of starch grains in the basal part was higher than that in the middle part and the top part. At stage 4, the nectaries still contained an abundance of starch grains. From stage 5 to stage 6, the starch grains decreased sharply. Although there were still a few starch grains in the middle and top parts, the starch grains were thoroughly degraded in the basal part. Therefore, the nectaries in *L. tulipifera* are starch-storing nectaries because they exhibit a growth and decline in starch. During stage 1 and stage 2, the nectaries accumulated starch. Then, starch production reached a peak at stages 3 and 4, which indicated that the nectaries were mature and that the starch was beginning to transform into nectar. These results were consistent with the morphological changes in the stomas. The changes in surface morphology and starch in the nectaries and the middle and top parts from stage 2 to stage 5 are vital to nectary development, nectar secretion and color change. Therefore, these petal parts were subject to detailed investigations via RNA-seq analysis.

### RNA-seq analysis of clean data in *L. tulipifera*

**Fig. 4:**
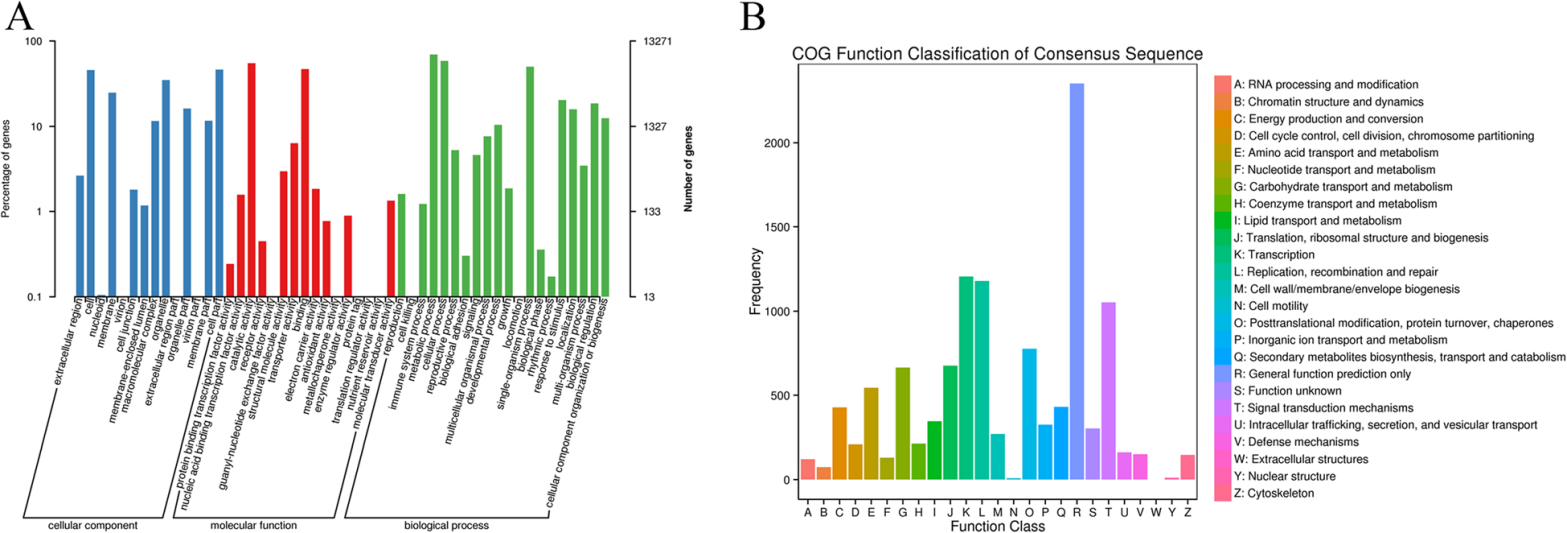
GO classification (**a**) and clusters of orthologous genes (**b**) of all identified genes

To explore the molecular mechanisms of nectary development and nectar secretion and the difference between the middle and top parts of the petals in *L. tulipifera*, RNA-seq analysis was performed with the Illumina Nova platform and 125-bp paired-end reads. Four samples were prepared from *L. tulipifera*: the middle and top parts at stage 2 (immature nectary) and the middle parts at stage 3 (mature nectary) and stage 5 (postsecretory nectary) (Table 1). RNA-seq analysis was performed with three biological replicates for each sample, and 12 libraries were generated in total. After removing low-quality reads, 115.26 Gb of clean data was obtained. The average number of reads per library was over 25 millions, and the Q30 base quality was no less than 93.85% (Table 1). The RNA-seq reads were mapped with onto *L. chinense* reference genome (NCBI, Bio Project, PRJNA418360) using HISAT2 with 71.02–79.77% efficiency for each sample. In total, 24,072 genes were identified, and 21,853 (90.78%) genes were annotated by BLAST for COG (7961), GO (13,271), KEGG (8986), Swiss-Prot (15,617), eggNOG (21,532), and NR (21,813) annotation. As shown in Fig. 4, the GO classification included biological process (30,021 genes), cellular component (12,643 genes), and molecular function (19,439 genes) (Fig. 4a). The COG classification included 25 functional categories, including transcription (1207 genes, 15.16%), replication, recombination and repair (1180 genes, 14.82%), and signal transduction (1053 genes, 13.23%) (Fig. 4). Furthermore, the annotated genes were enriched in 128 KEGG pathways. The top five enriched pathways were ribosome, plant-pathogen interaction, spliceosome, RNA transport and plant hormone signal transduction.

### Identification of DEGs in *L. tulipifera*

**Fig. 5:**
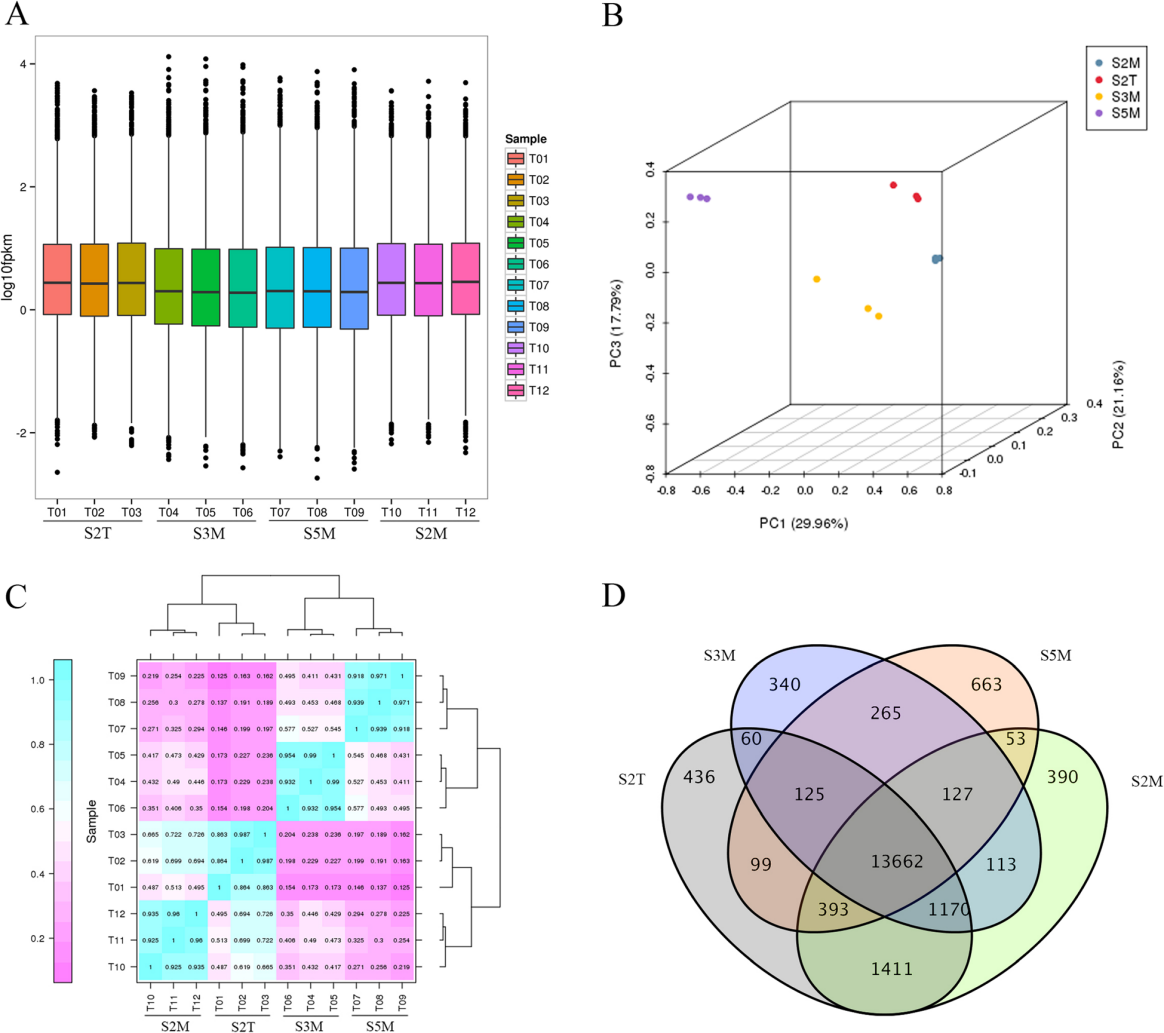
The analysis of the expressed genes. **a** Boxplot of the gene expression distribution for each sample; **b** Principal component analysis; **c** Correlations between two samples; **d** Venn diagram of four samples

To identify DEGs between different samples, we first evaluated gene expression levels based on a fragments per kilobase of transcript per million mapped reads (FPKM) threshold, using Cufflinks (v2.2.1) to measure the gene expression distribution for each sample (Fig. 5a). Principal component analysis was also performed and revealed that the 12 samples could be distinctly assigned to four groups: S2T, S2M, S3M, and S5M (Fig. 5b). Additionally, we analyzed the expressed genes in the four groups, and a Venn diagram showed the distribution of specific genes (436, 390, 340, and 663 expressed genes in S2T, S2M, S3M, and S5M, respectively) and shared genes (13,622 expressed genes) (Fig. 5d). Then, we uesd Pearson’s correlation coefficient (R^2^) as an index to assess the correlations between biological replicates (Fig. 5d). As Fig. 5 shows, the replicate samples were strongly correlated.

**Fig. 6:**
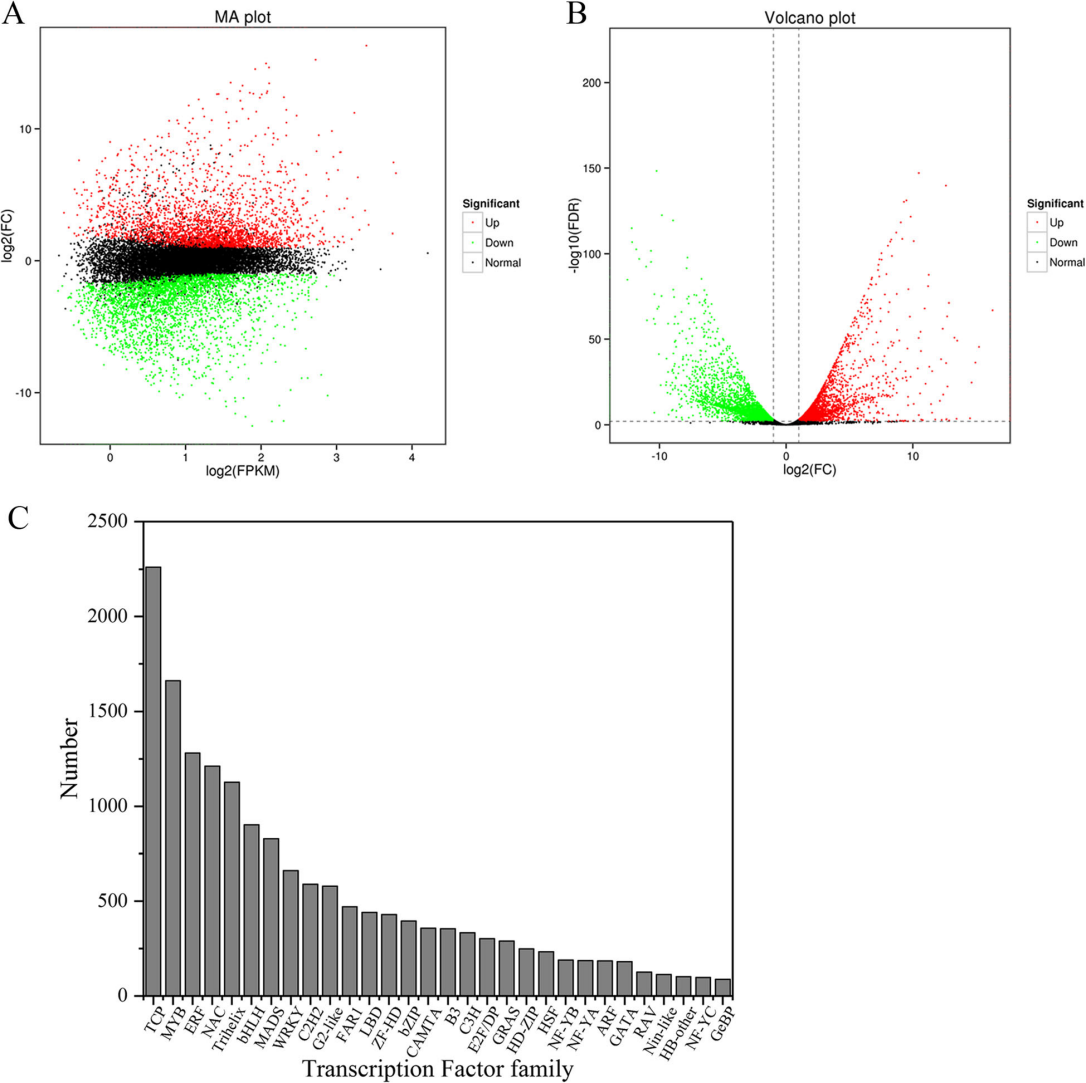
MA (**a**) and volcano plots (**b**) of RNA-seq data of the S2M_vs_S5M pairwise comparison; **c** The top 30 TF families were identified among the annotated DEGs

Then, DEGs were identified using an FC ≥2 and an FDR < 0.01 as criteria in each pairwise comparison. In total, the RNA-seq data were divided into six different pairwise comparisons, and 26,955 DEGs were identified, including 12,556 upregulated genes and 14,399 downregulated genes (Table 2). The DEGs were annotated using the COG (10,401 DEGs, 38.53%), GO (16,696 DEGs, 61.94%), eggNOG (25,586 DEGs, 94.92%), NR (25,747 DEGs, 95.52%), KEGG (10,207 DEGs, 37.87%) and Swiss-Prot (19,522 DEGs, 72.42%) databases (Table 3). The MA and volcano plots of each pairwise comparison clearly showed the distribution of upregulated and downregulated genes and their FCs (Fig. 6a, b). Consistent with the highly significant role of TFs during plant development and growth, we identified 56 major TF families by analyzing all annotated DEGs (Fig. 6c). The figure shows the top 30 TF families, mainly including the Teosinte branched1/Cincinnata/proliferating cell factor (*TCP*, 2261 DEGs), *MYB* (1661 DEGs), *ERF* (1281 DEGs), *NAM*/*ATAF1/CUC2* (*NAC*, 1211 DEGs), *Trihelix* (1126 DEGs), Basic helix-loop-helix (*bHLH*, 902 DEGs), and *MADS* (828 DEGs) TF families. Among these TF families, *TCP*, *ERF* and *NAC* are plant specific [42–44]. These TF families are widespread in many plant species and are involved in the control of plant development, senescence, abiotic and biotic stress responses, and floral bilateral symmetry. Noticeably, many genes encode members of the *Trihelix* TF family, which has gained interest only in recent years. This is the first report of the *Trihelix* TF family in *L. tulipifera* at the transcriptome level.

**Table 2:** Numbers of DEGs in each developmental stage and tissues

	S2T	S2M	S3M	S5M
S2T	–	473 + **142**	2558 + **1802**	3304 + **2732**
S2M	**473** + 142	–	2704 + **2112**	3645 + **3179**
S3M	**2558** + 1802	**2704** + 2112	–	2046 + **2258**
S5M	**3304** + 2732	**3645** + 3179	**2046** + 2258	–

Numbers in bold and in normal text indicate the number of upregulated and downregulated genes when the sample in bold text is compared with the sample in normal text, respectively

**Table 3:** Annotation of DEGs in six pairwise comparisons

DEG Set	DEG Number	COG	GO	KEGG	NR	Swiss-Prot	eggNOG
S3M_vs_S5M	4304	1714	2729	1649	4126	3180	4101
S2M_vs_S2T	615	243	435	251	587	488	587
S2M_vs_S3M	4816	1836	2944	1816	4599	3426	4561
S2M_vs_S5M	6824	2588	4138	2578	6495	4885	6452
Total	26,955	10,401	16,696	10,207	25,747	19,522	25,586

### Analysis of DEGs involved in nectary development and nectar secretion in *L. tulipifera*

**Fig. 7:**
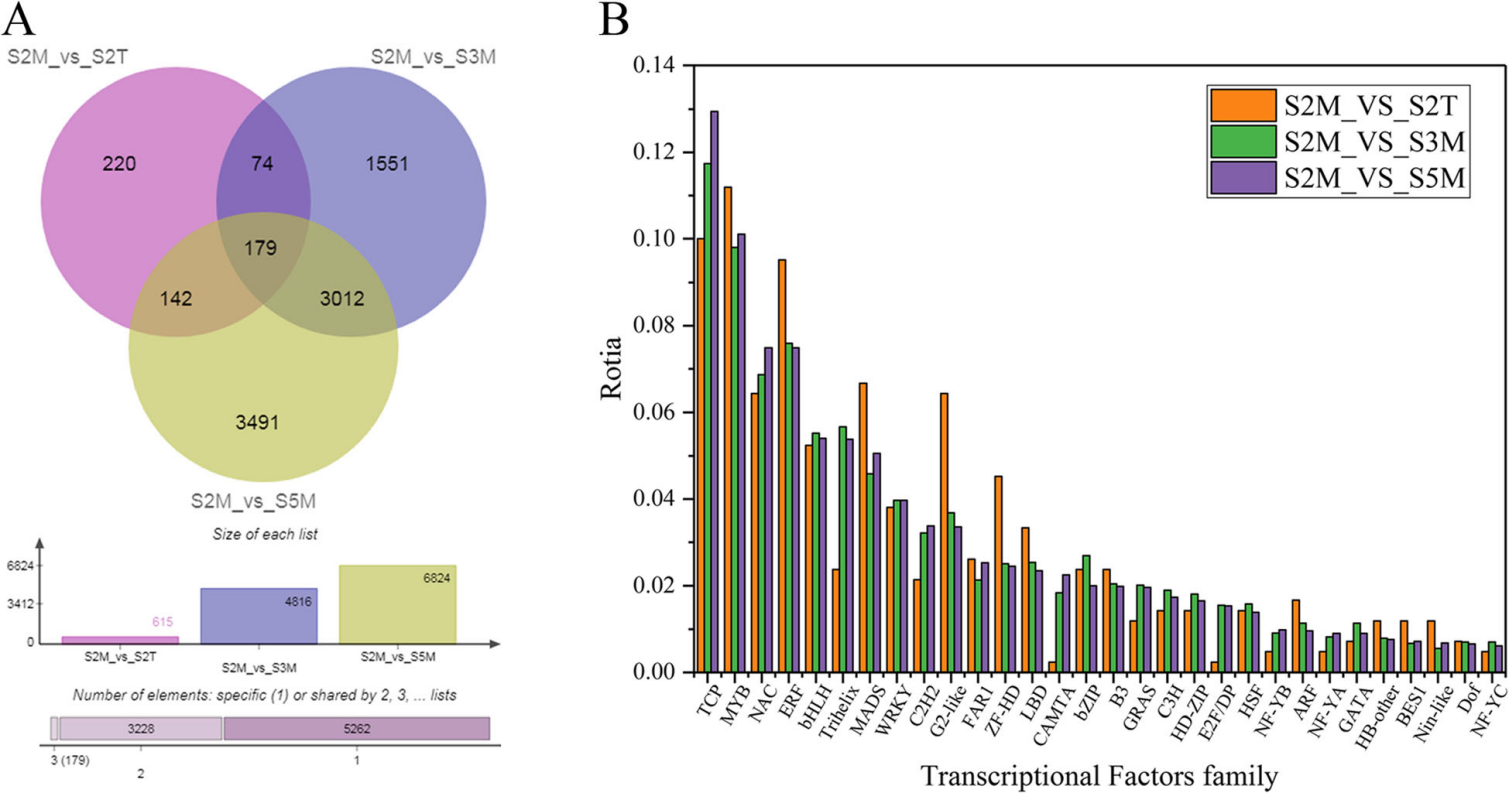
The analysis of DEGs and TF families among the three comparisons. **a** Venn diagram showing the number and overlapping events of DEGs; **b** The dynamic changes in TF families during nectary development

Based on the above results, the nectaries are located on the petals in *L. tulipifera*. It is very interesting that the color of the nectaries undergoes evident changes during their development (Fig. 1). Three pairwise comparisons were performed to further analyze the regulatory mechanisms of and key genes involved in nectary development, nectar secretion and color change in *L. tulipifera*, and S2M was used as a control and compared with S2T, S3M and S5M. A Venn diagram showing the transcript levels for the three pairwise comparisons was generated to further explore the number and distribution of overlapping genes among the three comparisons, (Fig. 7a). In S2M_vs_S2T, 615 DEGs were identified, with 473 upregulated genes and 142 downregulated genes; in S2M_vs_S3M, 4816 DEGs were identified, with 2112 upregulated genes and 2704 downregulated genes; and in S2M_vs_S5M, 6824 DEGs were identified, with 3179 upregulated genes and 3635 downregulated genes (Table 4, Fig. 7a). In addition, we analyzed the changes in TF family dynamics in the three comparisons during nectary development and nectar secretion (Fig. 7b). The results for S2M_vs_S3M and S2M_vs_S5M were similar. When S2M_vs_S2T was compared to the other pairwise comparisons, the number of DEGs encoding the *TCP*, *Trihelix*, *C2H2*, *CAMTA* and *E2F/DP* TF families notably declined, and the number of DEGs of the *ERF*, *MADS*, *Golden2*-like (*G2*-like), and *Zinc Finger Homeodomain* (*ZF-HD*) TF families increased.

**Table 4:** Numbers of annotated DEGs in three pairwise comparisons

DEG Set	Total	COG	GO	KEGG	NR	Swiss-Prot	eggNOG
S2M_S2T	587	243	435	251	587	488	587
S2M_S3M	4607	1836	2944	1816	4599	3426	4561
S2M_S5M	6499	2588	4138	2578	6495	4885	6452

**Fig. 8:**
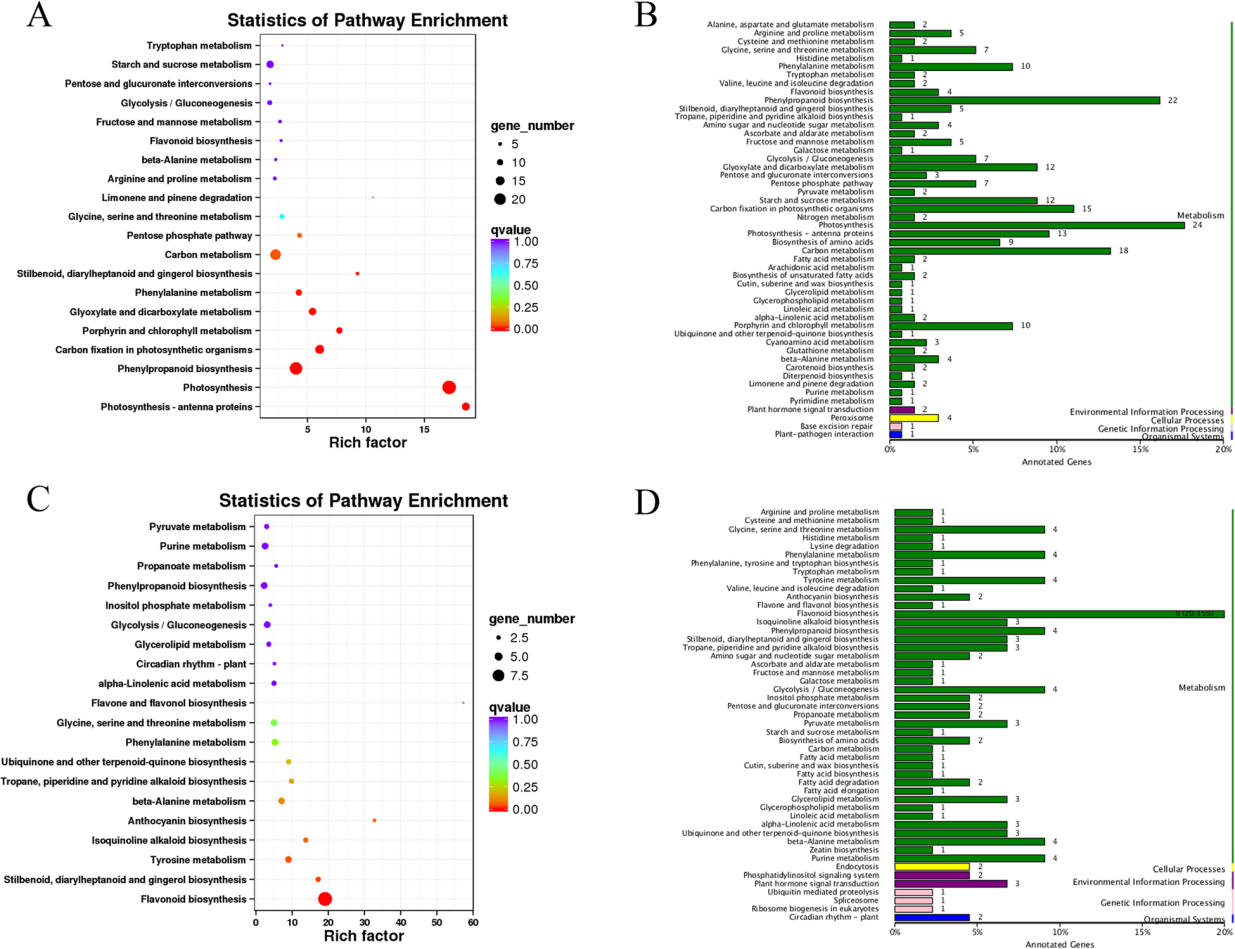
The KEGG pathway categories and enrichment factor analysis of S2M_vs_S2T DEGs. **a**, **b** Upregulated genes; **c**, **d** Downregulated genes

The S2M_vs_S2T comparison produced the smallest number of DEGs (in total 615 DEGs), and 95.45% (587 DEGs) of them were annotated. We analyzed the KEGG pathways of 415 upregulated and 135 downregulated annotated DEGs and chose the 20 smallest Q values to draw scatter plots of pathway enrichment (Fig. 8). The upregulated genes were functionally assigned to 50 biological pathways including photosynthesis (24 DEGs, 5.78%), phenylpropanoid biosynthesis (22 DEGs, 5.30%), and carbon metabolism (18 DEGs, 4.34%) and the downregulated genes were mainly assigned to flavonoid biosynthesis (9 DEGs, 6.67%). S2T and S2M corresponded to the top and middle parts of the same petal at stage 2, so the pairwise comparison produced fewer DEGs. Although the S2T and S2M parts were the same petal, the former was green, while the latter was green-white. The KEGG pathway analysis showed that the DEGs of S2T were significantly closely related to photosynthesis, which was consistent with the color. Considering that the DEGs of S2M were significantly enriched in flavonoid biosynthesis, we speculate that the difference in the color between the top and middle parts of the petals is related to flavonoids (Fig. 11).

**Fig. 9:**
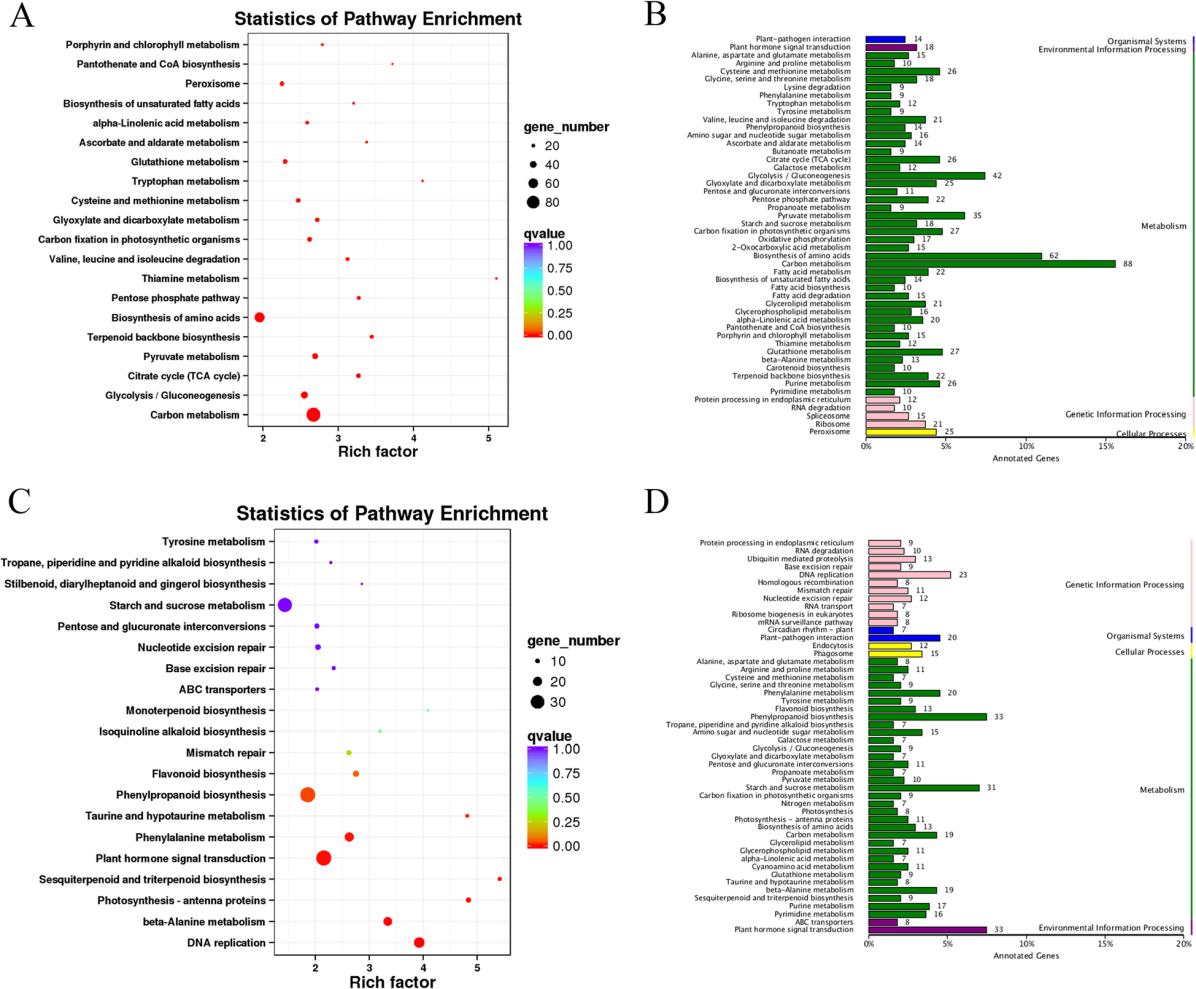
The KEGG pathway categories and enrichment factor analysis of S2M_vs_S3M DEGs. **a**, **b**: Upregulated genes; **c**, **d** Downregulated genes

Then, we also functionally analyzed the KEGG pathways of S2M_vs_S3M DEGs. A total of 4607 (95.66%) DEGs were annotated, including with 2021 upregulated and 2586 downregulated genes. The upregulated genes were mainly classified as being involved in carbon metabolism (88 DEGs, 4.35%), biosynthesis of amino acids (62 DEGs, 3.07%), glycolysis/gluconeogenesis (42 DEGs, 2.08%) and terpenoid backbone biosynthesis (22 DEGs, 1.09%) (Fig. 9b). Thiamine metabolism, tryptophan metabolism and terpenoid backbone biosynthesis were significantly enriched based on pathway enrichment factor analysis (Fig. 9a). The downregulated genes were mainly classified as being related to phenylpropanoid biosynthesis (33 DEGs, 1.28%), plant hormone signal transduction (33 DEGs, 1.28%), starch and sucrose metabolism (31 DEGs, 1.20%) and flavonoid biosynthesis (13 DEGs, 0.50%) (Fig. 9d). According to analysis of starch metabolism during nectary development, the starch peaked at stage 3. The S3M DEGs were significantly assigned to carbon metabolism, which was consistent with the PAS results. The terpenoid backbone biosynthesis pathway is also notable; we think that the terpenoid backbone biosynthesis pathway may be related to floral volatiles, and terpenoids are an essential ingredient of volatiles in *L. tulipifera* considering that the flower is prepared for anthesis at stage 3.

**Fig. 10:**
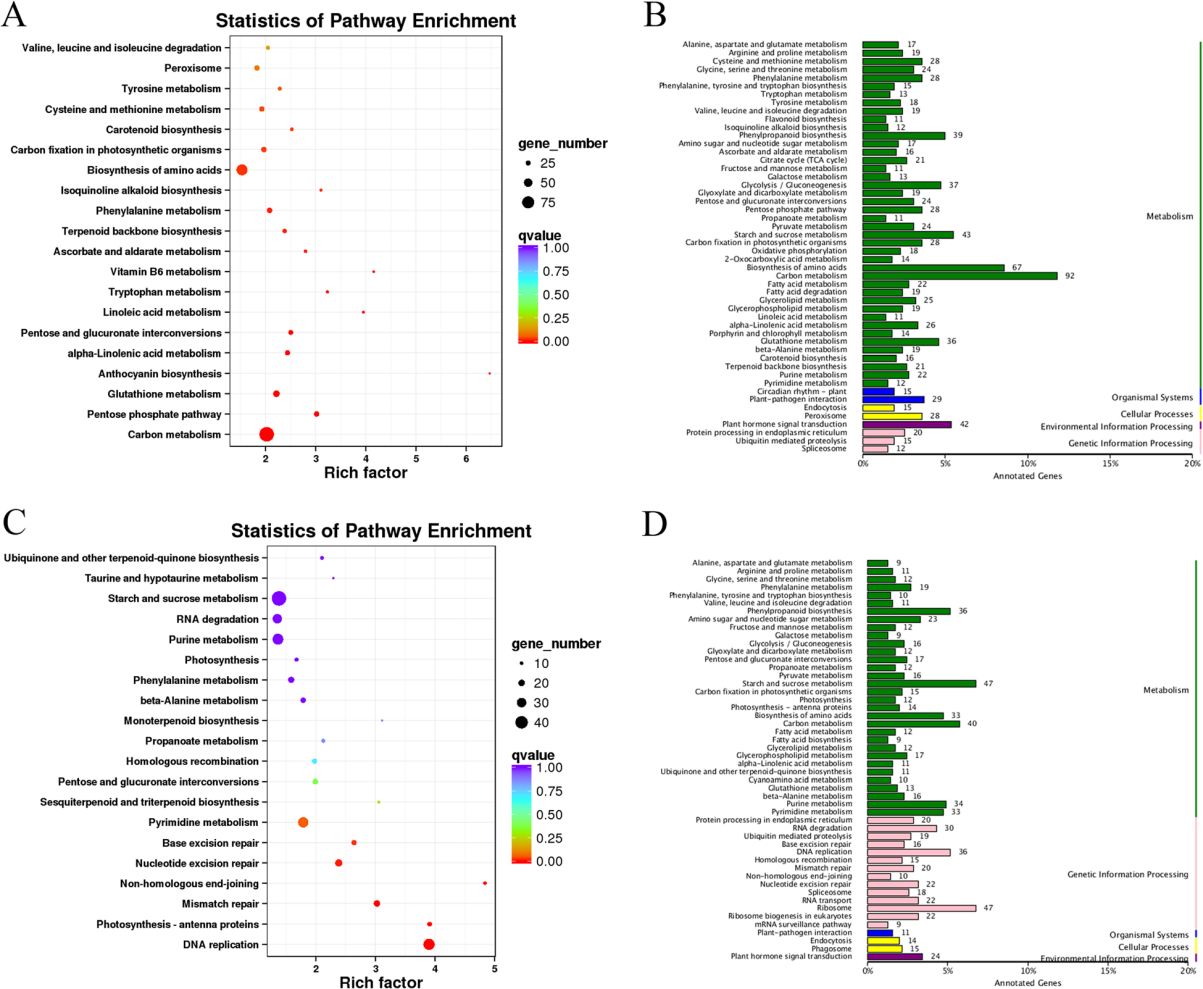
The KEGG pathway categories and enrichment factor analysis of S2M_vs_S5M DEGs. **a b** Upregulated genes; **c**, **d** Downregulated genes

**Fig. 11:**
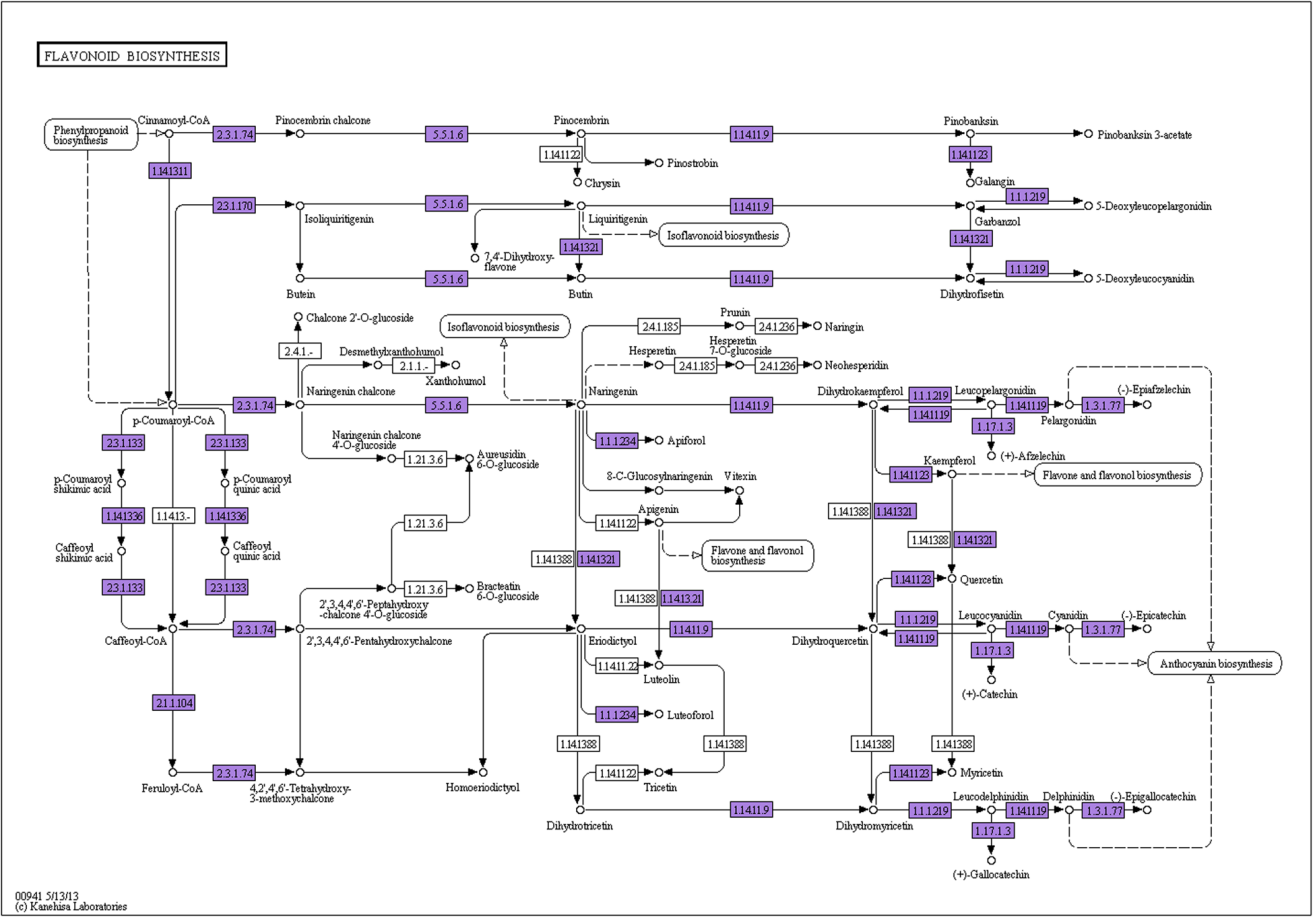
There are 55 genes involved in flavonoid biosynthesis in *L. tulipifera*

Moreover, we analyzed the annotated DEGs from the S2M_vs_S3M pairwise comparison. A total of 6499 (95.24%) annotated DEGs, including 3058 upregulated and 3441 downregulated genes were identified. The upregulated genes were classified as being involved in carbon metabolism (92 DEGs, 3.01%), biosynthesis of amino acids (67 DEGs, 2.19%), starch and sucrose metabolism (43 DEGs, 1.41%) and phenylpropanoid biosynthesis (39 DEGs, 1.28%) (Fig. 10b). The enrichment factor of anthocyanin biosynthesis was more significant than that of other pathways (Fig. 10a). The downregulated genes were classified as being related to starch and sucrose metabolism (47 DEGs, 1.37%), ribosome (47 DEGs, 1.37%), and carbon metabolism (40 DEGs, 1.16%) (Fig. 10d). S5M is an important stage for nectar secretion; therefore, the DEGs were involved in carbon, starch and sucrose metabolism. The middle part of the petals was orange-red at stage 5, and anthocyanin biosynthesis was highly significant; thus, anthocyanin may explain the difference between S2M and S5M (Fig. 11).

### RT-qPCR analysis of nectary-related genes

**Fig. 12:**
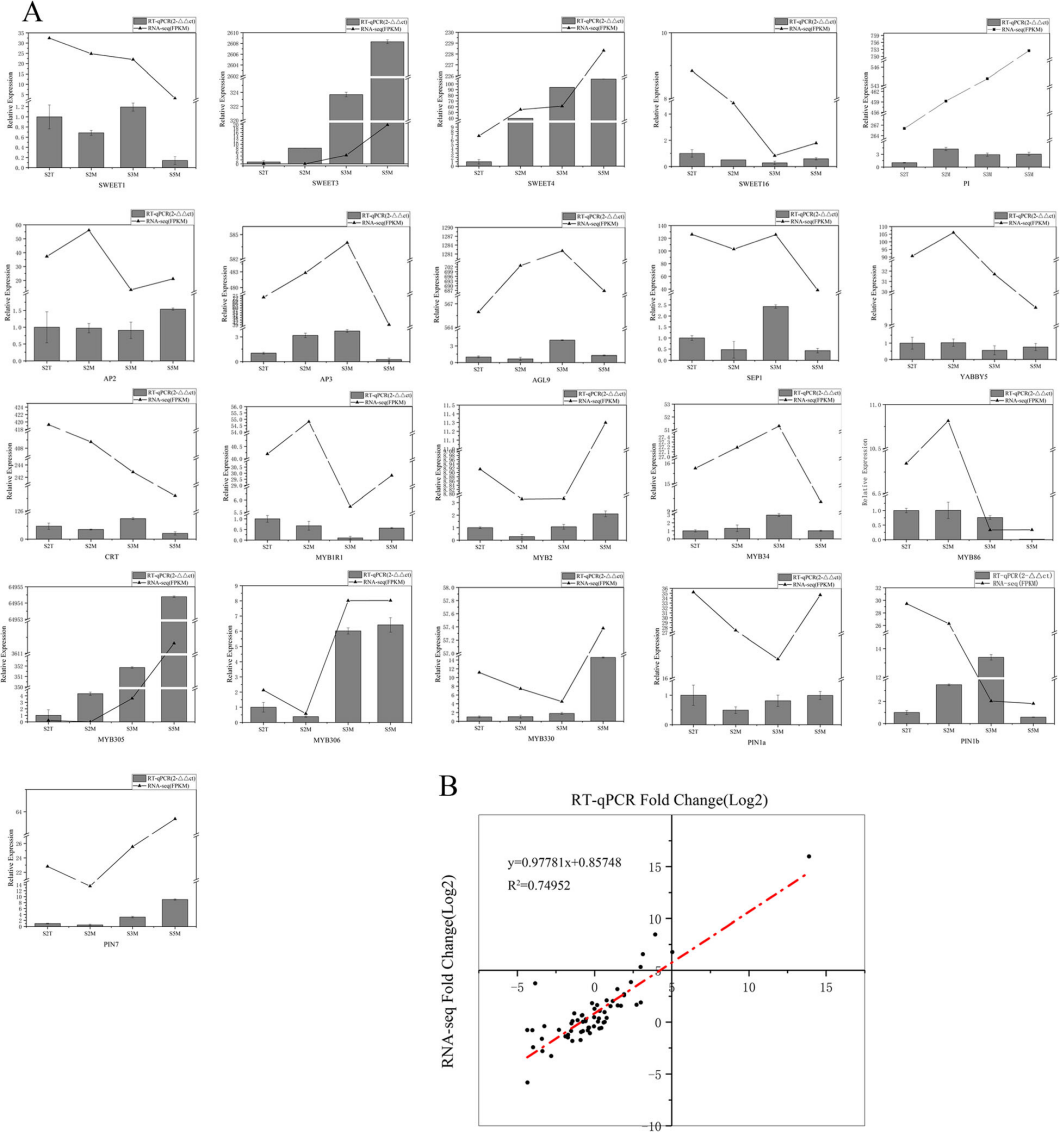
RT-qPCR validations of RNA-seq data. **a**: Expression profiling of 21 candidate genes involved in nectary development and nectar secretion in *L. tulipifera*; **b**: Scatter plot showing the correlation between RNA-seq and RT-qPCR

To calculate the accuracy of RNA-seq, we chose several DEGs related to nectary development and nectar secretion. Nectary development is primarily regulated by *CRABS CLAW-like* (*CRC*), *BLADE-ON-PETIOLE-like* (*BOP*), and members of the *MADS-box* family including *APETALA* (*AP*), *PISTILLATA* (*PI*), *AGAMOUS* (*AG*), and *SEPALLATA* (*SEP*), and nectar secretion is regulated by *SWEET-like*, *CELL WALL INVERTASE-like* (*CWINV*), *MYB-like*, Calreticulin (*CRT*), *Nectarin1/3*, and auxin efflux transporter *PIN-like* according to several studies of *A.thaliana* and *Nicotiana tabacum*. We identified 21 DEGs by the functional prediction of annotated genes from the RNA-seq data, including *LtYABBY5*, *LtCRT*, *LtSEP1*, *LtAP2*/*3*, *LtAGL9*, *LtPI*, *LtSWEET1*/*3*/*4*/*16*, *LtPIN1a*/*b*, *LtPIN7*, *LtMYB2*/*34*/*86*/*305*/*306*/*330*, and *LtMYB1R1*. The 21 candidate genes were analyzed to verify the expression patterns of the RNA-seq data with RT-qPCR analysis. The expression profiles were analyzed by relative expression (RT-qPCR) and FPKM (RNA-seq) values. Then, the correlation between RNA-seq and RT-qPCR data was measured by a scatter plot of log2-FC values (Fig. 12a). The differential expression profile of DEGs was consistent between the RNA-seq and RT-qPCR data and showed a positive correlation coefficient (R^2^ = 0.74952, *P* < 0.01) (Fig. 12b). Although 1 gene (4.76%, *LtPIN1b*) was significantly different in the expression profile, 16 genes (76.19%) displayed similar expression profiles when the RT-qPCR data were compared with the RNA-seq data. The MADS-box family mainly regulates the early development of floral organs in flowering plants; thus, we found that the *PI*, *AP2*, *AP3*, *AGL9* and *SEP1* genes showed low expression levels in mature petals of *L. tulipifera*. Strikingly, the selected genes from the *MYB*-like and *SWEET*-like families exhibited high expression in S3M and S5M in *L. tulipifera*, especially the *MYB305* and *SWEET3* genes. These results suggest that these genes may be involved in regulating nectar secretion in *L. tulipifera*.

## Discussion

### Nectary development and nectar secretion in *L. tulipifera*

The nectary is a multicellular glandular structure that may occur on vegetative or reproductive organs and secretes nectar and sweet solutions [45]. Research on nectary structure and classification has been performed for several species but is still lacking in *Liriodendron* species. *Liriodendron* species have a low seed set and tend to be endangered, especially *L. chinense*. *L. tulipifera* has a higher seed set than *L. chinense*, which is closely related to the more colorful petals and more plentiful nectar of the former. *L. tulipifera* is a nectariferous and insect-pollinated plant, and its nectar is the major attractant for insects. Therefore, it is urgent to thoroughly understand the nectary classification, development and secretion process in *L. tulipifera* and explore why nectar production differs significantly in *Liriodendron* plants. In this study, we evaluated nectary surface cells, stomas, and starch metabolism in detail. The results show that nectaries in *L. tulipifera* are floral, petal and starch-storing nectaries. The nectaries of several *Magnolia* species are stigmatic or carpel nectaries, and *M. stellata* nectar secretion occurs by epithelial ovarian nectaries in which the epidermis of the entire carpel is involved [46, 47]. Considering the differences in *Magnolia* and *Liriodendron* nectaries, we inferred that the nectaries of the different genera in Magnoliaceae may not be homologous or may have undergone differentiation or independent evolution [2, 48]. The floral nectaries of many species form a protrusion of the organ that bears them, but we found that the nectaries in *L. tulipifera* are flush with the surface [49, 50]. The nectaries in *Coptis* and *Hydrastis* of Ranunculaceae, members of the basal eudicots, are located on the petals and flush with the surface, which are similar to the features of nectaries in *L. tulipifera* [51]. In monocots, the nectaries are septal nectaries and are exclusive to this lineage, and in core eudicots, intrastaminal nectaries are common, followed sequentially by gynoecial, extrastaminal, hypanthial, petal and sepal nectaries [48, 52]. Compared with the other nectary types, the petal nectaries in *Liriodendron*, *Coptis* and *Hydrastis* may have primitive characteristics.

In *L. tulipifera*, nectary color substantially changes from green to yellow to orange-red with development. Similarly, tobacco nectaries change from lime green to yellow to bright orange [48]. Moreover, the starch content in tobacco nectaries increases during development and degrades prior to anthesis, which is consistent with the trend in *L. tulipifera* [48]. Therefore, during future research on the molecular mechanisms and genetic transformation of nectary development and nectar secretion, we can use tobacco as an assistant material rather than other species.

### The vital genes involved in nectary development and nectar secretion in *L. tulipifera*

Nectaries are crucial for insect participation in pollination. However, the molecular mechanisms of nectary development and nectar secretion remain unclear and are a relatively new area of research. Only *CRC* and *BOP* have been found to regulate nectary development, and *CWINV4*, *MYB* and *PIN6* have been shown to be related to nectar secretion [11, 13, 15, 17, 20]. Moreover, there are few studies on the molecular mechanisms of nectary development and nectar secretion in *L. tulipifera*. Therefore, it is indispensable and urgent to conduct RNA-seq in order to provide significant insights into these processes. Based on stomal morphology and starch accumulation, immature, mature and postsecretory nectaries were chosen for RNA-seq analysis. In total, 26,955 DEGs and 56 TF families, including the *TCP*, *MYB*, *ERF*, *NAC*, *Trihelix*, *bHLH*, and *MADS* families, were identified. Combining the results of previous studies and our RNA-seq gene expression analysis, we detected 21 DEGs via expression profiling with RT-qPCR. *CRC* is a vital and conserved gene for nectary development in core eudicot species, and no evidence of its expression in basal eudicot nectaries has been found [2]. During our RNA-seq analysis, we also did not detect the expression of CRC in *L. tulipifera*, which was consistent with the findings of previous research [2].

The most dramatic change in ornamental tobacco nectaries is the change in color during nectary development, which is consistent with the change observed in *L. tulipifera* nectaries [48]. The color change from lime green to yellow to bright orange is related to the high concentration of β-carotene [53]. The color change among S2T, S2M, S3M and S5M in *L. tulipifera* was from green to yellow to orange-red. The pigments in plants mainly include carotenoids, flavonoids and betalains [54]. The three types of pigments are responsible for different colors. Flavonoids are a group of secondary metabolites belonging to the phenylpropanoids, and anthocyanins are a class of flavonoids [54, 55]. By analyzing the KEGG pathway enrichment of DEGs, we found that a total of 55 genes were enriched in phenylpropanoid biosynthesis, flavonoid biosynthesis, terpenoid backbone biosynthesis and anthocyanin biosynthesis in S2T, S2M, S3M and S5M in *L. tulipifera* (Fig. 11). These findings suggest that flavonoids are responsible for the yellow color in S2M and that the orange-red color is caused by the accumulation of anthocyanins in S5M in *L. tulipifera*. Phenylpropanoid, flavonoid and anthocyanin biosynthesis and metabolism are important for pigment studies in *L. tulipifera*. We found that the TF *MYB305* was strongly expressed in S5M in *L. tulipifera* via RT-qPCR analysis. *MYB305* is also highly expressed in tobacco nectaries and positively regulates flavonoid metabolism and nectar production [16, 17]. We speculate that *LtMYB305* is involved in nectary development and regulates flavonoid and nectar production in *L. tulipifera*. In future work, we will measure the anthocyanin content in different stages and identify the function of *LtMYB305* in nectary development, secretion and pigmentation.

The major component in nectar is sucrose, and sucrose efflux is mediated by SWEET proteins as a key step in phloem transport [56]. *AtSWEET11*,*12* have been confirmed to be responsible for sucrose efflux, and *SWEET9* is essential for nectar production and functions as an efflux transporter in *A. thaliana*, *Brassica rapa, Petunia hybrida* and *Nicotiana attenuata* [57, 58]. We analyzed the expression profiles of *LtSWEET1,3,4,16* by RT-qPCR. Notably, *LtSWEET3* was highly expressed in S5M and S3M. The expression profile of *LtSWEET4* was similar to that of *LtSWEET3*, but compared with that of the former, the expression of the latter was tenfold higher. The nectary morphology assessed via SEM analysis indicated that nectar was secreted in S3M and S5M. Moreover, based on KEGG pathway analysis, carbon metabolism and the starch and sucrose metabolism pathways were primarily enriched in S3M and S5M. Combining these results, we suggest that *LtSWEET3,4* are related to nectar secretion in *L. tulipifera*. The TFs *MYB21,24,57,305* are nectary-enriched genes in *Arabidopsis* and ornamental tobacco and regulate nectary development, flavonoid metabolism and nectar production [17, 18, 20]. *AtMYB1R1,2,34,86,305,306,330* are differentially expressed in nectaries. Among these TFs, *AtMYB305* attracts our attention because its expression is far higher than that of the other TFs. *LtMYB305* was mainly expressed in S3M and S5M, which was similar to the patterns observed for *AtMYB2,306,330* and *LtSWEET3,4*. In summary, the *SWEET* and *MYB* families are important for nectar secretion in *L. tulipifera*. These findings enrich the understanding of molecular networks and provide new insights into the mechanism of nectary development and nectar secretion in *L. tulipifera*. Next, we will focus on the function of *SWEET, MYB*, and *YABBY* family genes, especially *CRC*, *SWEET3*, and *MYB305*, to explore the molecular mechanisms of nectary development and secretion in *L. tulipifera*.

## Conclusions

In this study, we recorded changes in surface cells, stomas and starch during nectary development by SEM and PAS methods in *L. tulipifera*. The floral nectaries were located on the petals, were starch-storing nectaries and secreted nectar from stage 3 to stage 5. These findings not only expand our understanding of angiosperm nectaries but also strongly motivate RNA-seq analysis. We constructed 12 libraries and obtained 115.26 Gb of clean data that mapped to the *L. chinense* reference genome with 71.02–79.77% efficiency. According to KEGG pathway analysis, the flavonoid, phenylpropanoid, and anthocyanin biosynthesis and starch and sucrose metabolism pathways may be related to nectar secretion and pigment change. Additionally, we found that the *TCP*, *Trihelix*, *C2H2*, *ERF*, and *MADS* TF families undergo dynamic changes during nectary development. The *YABBY*, *SWEET* and *MYB* genes also have high expression in *L. tulipifera* nectaries, according to RT-qPCR validation.

## Supplementary information

**Additional file1: Table S1.** List of 21 DEGs primers used for RT-qPCR.

## Data Availability

All data generated or analyzed during this study are included in this published article and its additional files.

## Abbreviations

COG: Cluster Orthologous Groups
DEGs: Differentially expressed genes
FC: Fold change
FDR: False discovery rate
FPKM: Fragments per kilobase of transcript per million mapped reads
GO: Gene Ontology
KEGG: Kyoto Encyclopedia of Genes and Genomes
KOG: Eukaryotic Orthologous Groups
RSEM: RNA-seq by expectation maximization
RT-qPCR: Real-time quantitative PCR
SEM: Scanning electron microscopy
PAS: Periodic acid-Schiff techniques
TFs: Transcription factors
