# Supplementary material for: Transcriptomic and microstructural analyses in Liriodendron tulipifera Linn. reveal candidate genes involved in nectary development and nectar secretion
Source: BMC Plant Biol. 2019 Dec 2;19:531. doi: 10.1186/s12870-019-2140-0 (PMC6889543; doi:10.1186/s12870-019-2140-0)
Supplement: Supplementary file 1 — Additional file1: Table S1. List of 21 DEGs primers used for RT-qPCR. [file 12870_2019_2140_MOESM1_ESM.docx]

Table S1. List of 22 DEGs primers used for RT-qPCR

| Genes | Primers |
| --- | --- |
| *LtActin97* | F: TTCCCGTTCAGCAGTGGTCG |
|  | R: TGGTCGCACAACTGGTATCG |
| *LtSWEET3* | F: CGAAACCAATGCCGTTCTTCTCT |
|  | R: AATTCATGCCTTTCAATCTGTCCT |
| *LtPIN 7* | F: ATACATCACCACCCCTTCCCT |
|  | R: TGTTGCCACCGAGAATAAGTTCCA |
| *LtMYB306* | F: AGCCTTCTCTCCCATTGTCC |
|  | R: TGCCATAGCTTCTTACCTCCCT |
| *LtMYB34* | F: ACTGTTCCGATCCCGACGCTTG |
|  | R: ATATCATGGACACCGCCTGCT |
| *LtMYB2* | F: ACTCTCGGAAGACAGAATTTGGG |
|  | R: CCGGCATTTCTTTCATCTCAGGT |
| *LtPIN1a* | F: AAAAGGCCCAATGGACAGCAA |
|  | R: CTGCTCCTTTTCCCATCACCT |
| *LtSWEET4* | F: GTGTCCAGGTCCATACAA |
|  | R: AAGGTCCGATAGGTGAAC |
| *YABBY 5* | F: TCCTGCTTTCTCGCTTGCTCT |
|  | R: CACCGCGAGAATAATGTTGCAGA |
| *LtPI* | F: ATTGAAGTAAGATCGCTCCCCTG |
|  | R: CATGTCTGGCATCCGTTCGTT |
| *LtSWEET1* | F: CCCTGGCCGTCTTCTCCGTG |
|  | R: GGGCATGAACTCTACGCTCT |
| *LtMYB1R1* | F: AACTGCCACCTTCCAGACACC |
|  | R: ACAGTGATTATGCTCCCTCCC |
| *LtSEP1* | F: AGGTTGCACTCATCATCTTCTCT |
|  | R: GCGGGTCCAAATCTTCACCA |
| *LtAP2* | F: ATCATGTGGGATCTGAACGACT |
|  | R: TTCCTCGTGGTCGGATGCTT |
| *LtMYB330* | F: ATATCGCCATCTCCTACAC |
|  | R: CTGAAGGTGGATTGGATTG |
| *LtMYB305* | F: CTGGACTATGGAGGAAGAC |
|  | R: GTGTTGAAACGAGGCTATC |
| *LtMYB86* | F: AGGTTTTCCGCAGCAATAGCA |
|  | R: CGCTCCAATACAAACTCTTCCCT |
| *LtPIN1b* | F: CGAGTAATGAGAGCCGTAT |
|  | R: CAAGGTGAGTGATTGAGATG |
| *LtCRT* | F: ATCCAAACCACCGAAGACCAC |
|  | R: AGTGATTCTTTCCATTGCGTGT |
| *LtSWEET16* | F: GTTTCTCATCTATGCGCCTCCC |
|  | R: CGGCATGTATTCCACGCTCT |
| *LtAP3* | F: CTTGTTCACCTTCCATCTTC |
|  | R: TCATAATCCCACCTCTTCTC |
| *LtAGL9* | F: AGCAAATCAGATCAACCCGGAC |
|  | R: TGATTTTCCTGCACTCCTTCCT |
